# Return to work, work productivity loss and activity impairment in Chinese breast cancer survivors 12-month post-surgery: a longitudinal study

**DOI:** 10.3389/fpubh.2024.1340920

**Published:** 2024-02-23

**Authors:** Danielle Wing Lam Ng, Serana Chun Yee So, Richard Fielding, Anja Mehnert-Theuerkauf, Ava Kwong, Dacita Suen, Ling Wong, Sara Wai Wun Fung, Oi Kwan Chun, Daniel Y. T. Fong, Sharon Chan, Alex Molasiotis, Winnie K. W. So, Wendy Wing Tak Lam

**Affiliations:** ^1^LKS Faculty of Medicine, School of Public Health, Centre for Psycho-Oncology Research and Training, The University of Hong Kong, Hong Kong, Hong Kong SAR, China; ^2^LKS Faculty of Medicine, Jockey Club Institute of Cancer Care, The University of Hong Kong, Hong Kong, Hong Kong SAR, China; ^3^University Medical Center Leipzig, Department of Medical Psychology and Medical Sociology, The University of Leipzig, Leipzig, Germany; ^4^LKS Faculty of Medicine, School of Clinical Medicine, Department of Surgery, The University of Hong Kong, Hong Kong, Hong Kong SAR, China; ^5^Department of Surgery, Tung Wah Hospital, Hospital Authority, Hong Kong, Hong Kong SAR, China; ^6^Department of Surgery, Kwong Wah Hospital, Hospital Authority, Hong Kong, Hong Kong SAR, China; ^7^LKS Faculty of Medicine, School of Nursing, The University of Hong Kong, Hong Kong, Hong Kong SAR, China; ^8^Department of Surgery, United Christian Hospital, Hospital Authority, Hong Kong, Hong Kong SAR, China; ^9^School of Nursing, Hong Kong Polytechnic University, Hong Kong, Hong Kong SAR, China; ^10^College of Arts, Humanities and Education, University of Derby, Derby, United Kingdom; ^11^The Nethersole School of Nursing, The Chinese University of Hong Kong, Hong Kong, Hong Kong SAR, China

**Keywords:** return to work, employment, work productivity, activity impairment, breast cancer, work condition, survival analysis

## Abstract

**Introduction:**

Existing evidence of returning-to-work (RTW) after cancer comes predominately from Western settings, with none prospectively examined since the initial diagnostic phase. This study prospectively documents RTW-rate, time-to-RTW, work productivity loss, and activity impairment, within the first-year post-surgery among Chinese women with breast cancer (BCW) and identify potential causal co-variants.

**Methods:**

This observational longitudinal study followed 371 Chinese BCW who were employed/self-employed at the time of diagnosis at 4-week post-surgery (baseline). RTW-status and time-to-RTW were assessed at baseline (T1), 4-month (T2), 6-month (T3), and 12-month (T4) post-baseline. WPAI work productivity loss and activity impairment were assessed at T4. Baseline covariates included demographics, medical-related factors, work satisfaction, perceived work demand, work condition, RTW self-efficacy, B-IPQ illness perception, COST financial well-being, EORTC QLQ-C30 and QLQ-BR23 physical and psychosocial functioning, and HADS psychological distress.

**Results:**

A 68.2% RTW-rate (at 12-month post-surgery), prolonged delay in RTW (median = 183 days), and significant proportions of T4 work productivity loss (20%), and activity impairment (26%), were seen. BCW who were blue-collar workers with lower household income, poorer financial well-being, lower RTW self-efficacy, poorer job satisfaction, poorer illness perception, greater physical symptom distress, impaired physical functioning, and unfavorable work conditions were more likely to experience undesired work-related outcomes.

**Discussion:**

Using a multifactorial approach, effective RTW interventions should focus on not only symptom management, but also to address psychosocial and work-environmental concerns. An organizational or policy level intervention involving a multidisciplinary team comprising nurses, psychologists, occupational health professionals, and relevant stakeholders in the workplace might be helpful in developing a tailored organizational policy promoting work-related outcomes in BCW.

## 1 Introduction

Breast cancer affects women worldwide, with a 2020 incidence of 2.26 million cases (1–3). Of these women ~67% were aged <65, with 56% being between 40 and 64 years old, considered the prime working years (4, 5). Increased incidence, improving 5-year survival (~80% in developed countries) (1) and extending retirement ages (6), mean growing numbers of working-age women are diagnosed with breast cancer (BCW) (5, 7). However, cancer patients have 1.6x and 1.3x higher risks for early retirement (8) and unemployment (9), respectively, and a lower incidence of reemployment than do healthy controls (10–12). Consequently, returning to work (RTW) and job retention are crucial unmet needs of this population, and of major concern in the field of cancer survivorship (5). For many, RTW after a cancer diagnosis and treatment is needed for financial stability and regaining normalcy, contributing to patients' wider recovery and quality of life (5, 11, 13–15). Societally, RTW for BCW has extrinsic economic value in terms of reduced direct and indirect medical and welfare costs, and from less disease-related productivity loss, especially in light of increasing female labor force participation (5, 11, 16, 17).

Data reporting global mean RTW rates for cancer patients (63.5%) ranges from 24 to 94%, depending on time since diagnosis (12). In Hong Kong, 6-month and 12-month post-cancer treatment RTW rates were 39 and 63%, respectively (17). A recent systematic review reported RTW rate among BCW within 12 months of diagnosis of between 43–93% (18), with a steadily rising trend observed over the survivorship trajectory (19), suggesting a majority eventually reentered the workforce. Yet, BCW may experience a prolonged delay in RTW compared to individuals with other forms of cancer (20). The average duration of work absence due to BC ranged from 86 to 349 days, with a median of 210 days (e.g. compared to 125 days for gynecological cancer patients) (18, 20). BCW's RTW is associated with work environment, physical and psychosocial functioning, educational level, personal finances, and illness perception, medical/treatment-related factors and social support (7, 11, 12, 15, 18–22). However, evidence is mixed regarding whether these determinants either facilitate or hinder RTW (15). Different regional contexts between studies may account for the reported conflicting evidence and varied RTW rates, reflecting differences in healthcare provision, employment policies, social security, and cultural perspectives on gender across regions (7, 17, 19, 21, 23). For example, in Hong Kong up to 120 paid sick leave days can be accumulated when supported by a valid medical certificate whereas in the United States no federal law guarantees the right to paid sick leave (17). Most existing RTW studies on BCW have studied Caucasian populations in western cultural settings post-treatment or during survivorship (21). Even among the scant evidence on East Asian populations (22, 24, 25), the RTW rates varied across countries; 37%-59% in Korea (22, 26), 41% in Malaysia (25), and 21–33% in China (27, 28). More importantly, there are no prospective studies following BCW from the initial diagnostic stage of the cancer journey to RTW and, thereafter, productivity in work, indicating an important gap in the RTW literature. Understanding BCW's employment experiences in different setting and tracking their RTW status longitudinally from the immediate aftermath of primary surgery more comprehensively illuminates the entire decision-making processes in RTW, clarifying associated long-term facilitators and barriers, enabling efficacious tailored interventions or supportive resources to be developed, and refining relevant laws and regulations to meet specific needs and promote early RTW, ultimately improving rehabilitation outcomes for this population (15, 17, 22). In this regard this study significantly advances the existing literature.

Following successful work resumption, BCW face employment-related challenges, including activity impairment and work productivity loss (absenteeism: time lost from work, and presenteeism: reduced work performance) (12), potentially preceding work cessation (17, 22). Greater activity impairment and work limitation, and poorer work performance, is reported by BCW than by healthy controls (12, 19, 29). However, we lack empirical evidence that disentangles whether BC and its treatments affect activity impairment and longer-term work productivity loss, and predictors thereof.

RTW after cancer is a complex process requiring the consideration of multiple factors (5). Mehnert (12) identified six influences on work-related outcomes: (i) demographic factors; (ii) impairments to health status such as physical symptoms; (iii) psychosocial factors like psychological well-being and social support; (iv) motivational factors like work satisfaction; (v) work-related factors like job type, work environment, employer accommodation, and relationship with co-workers; and (vi) work-related interventions like counseling and vocational training services. Given the varying regional contexts that may shape these influences, the next question is if this European model of BCW survivorship is applicable to Chinese BCW. For example, legal protection, medical costs and employer accommodation of employee needs, and perceptions of sick roles may vary, affecting both RTW delay and productivity.

Here we prospectively examine work-related outcomes (RTW rate, time to RTW, work productivity loss, and activity impairment) among Chinese BCW in the first year following primary surgery and identify factors predicting these outcomes. We hypothesized that certain sociodemographics (older age, lower educational attainment, being married, and higher personal financial toxicity), medical factors (cancer stage and treatment type), impaired physical and psychosocial functioning, lower work satisfaction and RTW self-efficacy, and unfavorable work-related factors (higher work demand and poorer work conditions) would be associated with poorer work-related outcomes. Negative illness perceptions affecting the “sick role” of cancer patients may hinder RTW and also lead to a greater loss of work productivity (17, 19), so illness perception was also assessed.

## 2 Materials and methods

### 2.1 Participants and setting

Following ethical approval (ref: UW 17-527), the recruitment was conducted at three dedicated breast centers in government-funded public hospitals in Hong Kong between December 2018 and August 2021. Cantonese- or Mandarin-speaking Chinese patients who (i) were newly diagnosed with curable BC, (ii) were aged 18 or above, (iii) were in paid- or self-employment at the time of diagnosis, and (iv) had completed surgery as primary treatment within the past 4 weeks were eligible. Patients with metastatic BC and linguistic or intellectual difficulties were excluded.

Potential participants were identified by surgical oncologists and approached by a trained research assistant during their postsurgical follow-up consultation. After obtaining fully informed written consent from patients who agreed to participate in the study, patients completed a standardized face-to-face baseline questionnaire immediately (within 1-month post-surgery, T1) and three follow-up assessments at 4-month (T2), 6-month (T3), and 12-month (T4) post-baseline through phone interviews.

### 2.2 Measures

#### 2.2.1 Work-related outcomes

Return to work (RTW), defined as time to RTW after an absence from work due to cancer (i.e., paid and unpaid time off from work) (9, 30), was assessed from 1-month post-surgery onwards (T1-T4). At each assessment, patients were asked to indicate if they had taken time off since the first day of sick leave. Those who reported having RTW were asked to specify the date of RTW. Time to RTW was calculated as the number of days between the first day of sick leave and the first day of RTW, irrespective of any job nature changes (e.g., changed job, reduced working hours) (20, 21). For those not taking time off from work, the time of RTW was set to zero.

Work productivity loss and activity impairment were assessed at 12-month post-surgery (T4) using the Chinese version of the Work Productivity and Activity Impairment (WPAI) questionnaire (31). The 6-item WPAI measures work productivity loss (the extent to which current health condition induced work inability) and activity impairment (the extent to which current health condition affected regular activities other than job-related work). Items assessing work productivity loss are only relevant to those who have returned to work. Both work productivity loss and activity impairment were expressed in percentages, with higher values indicative of greater productivity loss and impaired functioning (31).

#### 2.2.2 Potential covariates

Potential covariates include: (i) work satisfaction, assessed using a 12-item work satisfaction scale (32); (ii) perceived job strain/work demand, assessed using a 10-item measures (32); (iii) perceived work condition, assessed in terms of unfavorable work conditions such as physically heavy work, incorrect one-side posture, and excessive demand, using an 8-item measure (32); (iv) RTW self-efficacy, assessed using the 11-item Return-to-Work Self-Efficacy scale (RTW-SE scale) (32); (v) cognitive and emotional representations of illness, assessed using the Chinese version of the Brief Illness Perception Questionnaire (B-IPQ) (33, 34); (vi) cancer-related financial well-being, as assessed by the 11-item Comprehensive Score for Financial Toxicity (COST) (35); (vii) physical and psychosocial functioning, assessed by the standard Chinese version of the European Organization Research Treatment Caner (EORTC) general Quality of Life questionnaire (QLQ-C30) and the breast cancer specific module (QLQ-BR23) (36, 37); (viii) psychological distress, assessed by the 14-item Hospital Anxiety and Depression Scale (HADS) (38); (ix) demographics including age, marital status, education level, occupation, health insurance, and social welfare allowance details, collected through self-report; and (x) medical data including diagnosis, cancer stage, and treatment received, retrieved from hospital medical records after the completion of the study. All of which otherwise were assessed only once at baseline.

### 2.3 Data analysis

Standard descriptive analyses assessed sample characteristics and the RTW rate at each assessment timepoint. Kaplan-Meier survival analysis estimated the mean time to RTW. Cox regression and linear regression analyses were performed to identify potential covariates of the RTW rates, time to RTW, T4 work productivity loss, and T4 activity impairment, respectively (9, 20, 23, 39). Each of the potential covariates was entered into the regression model individually. Significant variables achieving a p-value of <0.05 were subsequently included in a multivariate regression model (20).

### 2.4 Sample size calculation

Based on our previous pilot study of RTW and work productivity among BCW, we expected 65–70% of BCW to return to work after BC diagnosis. To examine the RTW at 1 year after breast cancer diagnosis, with a 5% error margin and a confidence level of 95%, a sample of 352 patients was needed. To examine the change in work productivity and activity impairment, a standardized effect size of 0.4 was expected (9). With 80% power and a 5% maximum false positive error rate, at least 89 patients were needed after adjusting for multiplicity by Bonferroni correction.

## 3 Results

### 3.1 Sample characteristics

**Figure 1:**
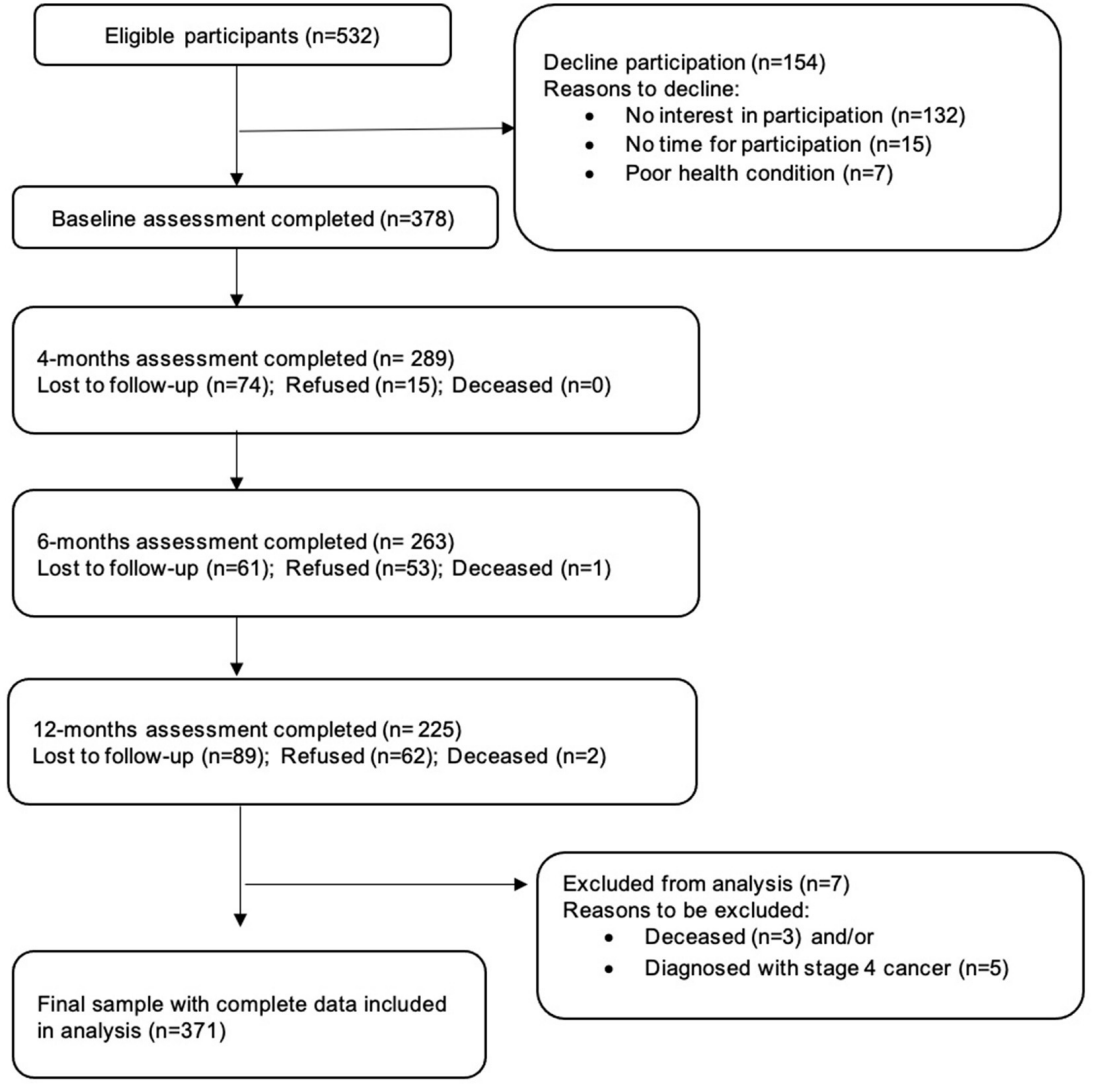
Sampling structure and attrition.

Of eligible patients, 71% (378/532) gave informed consent. Follow-up attrition ranged from 24%-40%. Seven participants died or/and were diagnosed with metastatic disease and excluded from analyses, leaving a final sample size of 371 (Figure 1). Excepting baseline QLQ-C30 nausea and constipation, neither demographic, clinical, nor baseline variables otherwise differentiated these two groups (Supplementary Table S1). The final sample had a mean age of 52.6 years. Most participants were married (60%), were at least secondary-level educated (85%), having a monthly household income <US$3,850 (57%), and undergoing active treatment at baseline (61%) (Table 1).

**Table 1:** Summary of demographic and clinical characteristics.

	**Participants (%) *n* = 371**
**Demographic characteristics**	
Age at diagnosis (year) ± standard deviation (SD)	52.62 ± 8.22 (range 26–72)
Time since cancer diagnosis (months) ± standard deviation (SD)	7.18 ± 11.04
**Marital status**	
Married/cohabited	222 (59.8)
Single/divorced/separated/widowed	147 (39.6)
Missing	2 (0.6)
**Educational level**	
No formal/primary education	52 (14.0)
Secondary/tertiary	317 (85.4)
Missing	2 (0.6)
**Job title**	
White collar	140 (37.7)
Blue collar	159 (42.9)
Professional, manager and self-employed	72 (19.4)
**Monthly household income (US$)**	
US$ < 1,280	62 (16.7)
US$ 1,280–3,850	150 (40.4)
US$ >3,850	147 (39.6)
Missing	12 (3.2)
**Clinical characteristics**	
**Stage**	
Stage 0	31 (8.4)
Stage I	126 (34.0)
Stage II	66 (17.8)
Stage III	32 (8.6)
Unknown	116 (31.1)
**Surgery type**	
Breast conserving surgery	165 (44.5)
Mastectomy or plus reconstruction	193 (52.0)
Missing	13 (3.5)
**Active treatment at baseline (T1)**	
Chemotherapy	75 (20.2)
Radiotherapy	36 (9.7)
Target therapy	41 (11.1)
Hormonal therapy	67 (18.1)
No active treatment	144 (38.8)
Missing	0 (0%)
Chemotherapy	70 (18.9)
Radiotherapy	34 (9.2)
Target therapy	41 (11.1)
Hormonal therapy	141 (38.0)
No active treatment	62 (16.7)
Missing	81 (23.5)
**Active treatment at FU2 (T3)**	
Chemotherapy	26 (7.0)
Radiotherapy	33 (8.9)
Target therapy	39 (10.5)
Hormonal therapy	157 (42.3)
No active treatment	82 (22.1)
Missing	93 (25.1)
**Active treatment at FU3 (T4)**	
Chemotherapy	3 (0.8)
Radiotherapy	6 (1.6)
Target therapy	20 (5.4)
Hormonal therapy	145 (39.1)
No active treatment	73 (19.7)
Missing	135 (36.4)
**Baseline predictors**	
Return-to-work self-efficacy (mean ± SD)	4.03 ± 0.75
COST-Financial well-being (mean ± SD)	22.75 ± 10.56
**IPQ-illness perception (mean** ±**SD)**	
Cognitive representations of illness	19.17 ± 7.12
Emotional representations of illness	10.69 ± 4.94
Work satisfaction (mean ± SD)	4.97 ± 1.08
Job strain (mean ± SD)	2.41 ± 0.84
**Work condition (mean** ±**SD)**	
Physically heavy work	2.76 ± 0.94
Incorrect one-sided posture	2.71 ± 0.88
Frequent/long hours in sitting position	2.83 ± 0.96
Wetness, coldness, and draft	2.31 ± 0.80
Rationalization and restructuring	2.12 ± 0.75
Implementation of new technologies	2.25 ± 0.83
Excessive demands	2.66 ± 0.79
Satisfying work nature	3.20 ± 0.64
QLQ-C30 global health status (mean ± SD)	61.10 ± 17.92
Physical functioning	74.00 ± 19.49
Role functioning	67.34 ± 27.08
Emotional functioning	72.06 ± 22.64
Cognitive functioning	76.01 ± 22.18
Social functioning	75.11 ± 25.57
**QLQ-C30 Symptoms scales (mean** ±**SD)**	
Fatigue	35.34 ± 24.16
Nausea	5.39 ± 12.87
Pain	32.35 ± 24.70
Dyspnea	13.75 ± 23.37
Insomnia	32.35 ± 32.45
Appetite loss	13.84 ± 22.47
Constipation	6.83 ± 14.95
Diarrhea	6.68 ± 17.99
Financial difficulties	31.08 ± 32.52
**QLQ-BC23 functional scales (mean** ±**SD)**	
Body image	78.46 ± 25.62
Sexual functioning	92.73 ± 14.43
Sexual enjoyment	61.11 ± 23.12
Future perspective	47.57 ± 30.08
**QLQ-BC23 symptoms scales (mean** ±**SD)**	
Systematic therapy side effect	15.94 ± 15.13
Breast symptoms	20.97 ± 20.30
Arm symptoms	26.68 ± 22.17
Upset by hair loss	38.61 ± 32.62
HADS Anxiety (mean ± SD)	5.40 ± 3.91
HADS Depression (mean ± SD)	4.71 ± 3.80

Participants could receive more than one active treatment at each assessment timepoint.

### 3.2 RTW rate

RTW rates were 23.2% (*n* = 86/371, 95%CI = 18.9–27.5%) at baseline, 49.6% (*n* = 141/284, 95%CI = 43.8–55.5%) at 4-month; 54.3% (*n* = 152/280, 95%CI = 48.4–60.2%) at 6-month, and; 68.2% (*n* = 165/242, 95%CI = 62.3–74.1%) at 12-month post-baseline.

Univariate analyses identified potential correlates of RTW status (Supplementary Table S2). In the final multivariable Cox regression model (Table 2), status with job title, monthly household income, receipt of post-operative chemotherapy and radiotherapy, RTW self-efficacy, B-IPQ illness perception, and work condition with long hours in sitting position were significant predictors of RTW. White collar (HR = 1.71, 95%CI = 1.19–2.45, *p* = 0.004) or professionals (HR = 1.62, 95%CI = 1.06–2.46, *p* = 0.025) vs. blue collar work, a higher monthly household income of >US$3,850 per month (<US$1,280: HR = 0.64, 95%CI = 0.41–0.98, *p* < 0.001; US$1,280–3,850: HR = 0.53, 95%CI = 0.39–0.73, *p* < 0.001), greater RTW self-efficacy (HR = 1.41, 95%CI = 1.20–1.87, *p* < 0.001), and more prolonged sitting at work (HR = 1.38, 95%CI = 1.17–1.62, *p* < 0.001) facilitated work resumption within 12 months after surgery. Chemotherapy (HR = 0.53, 95%CI = 0.39–0.70, *p* < 0.001), radiotherapy (HR = 0.45, 95%CI = 0.46–0.90, *p* = 0.010), and more negative cognitive representations of illness (HR = 0.97, 95%CI = 0.95–0.98, *p* < 0.001) hindered work resumption.

**Table 2:** Multivariate forward Cox regression on RTW status (*n* = 325/371).

	**β**	**SE**	**HR**	**95%CI**	***p*-value**
Job title					0.011^*^
**Blue collar (ref)**					
White collar	0.53	0.18	1.71	1.19–2.45	0.004^*^
Professional, manager and self-employed	0.48	0.21	1.62	1.06–2.46	0.025^*^
Monthly household income (US$)					< 0.001^**^
US$ < 1,280	−0.45	0.22	0.64	0.41–0.98	0.040^*^
US$ 1,280–3,850	−0.63	0.16	0.53	0.39–0.73	< 0.001^**^
**US$** >**3,850 (ref)**					
Chemotherapy	−0.64	0.15	0.53	0.39–0.70	< 0.001^**^
Radiotherapy	−0.45	0.17	0.64	0.46–0.90	0.010^*^
Return-to-work self-efficacy	0.32	0.082	1.38	1.17–1.62	< 0.001^**^
Cognitive representations of illness	−0.036	0.010	0.97	0.95–0.98	< 0.001^**^
**Work condition**					
Frequent/long hours in sitting position	0.21	0.085	1.24	1.05–1.46	0.012^*^
**Model statistics**					
X^2^	120.30				
*P*-value	0.011^*^				

^*^p-value < 0.05; ^**^p-value < 0.001.

### 3.3 Time to RTW

Using Kaplan-Meier survival analysis, the average time to RTW was 261 days, with a median of 183 days (Supplementary Figure S1).

Among correlates of time to RTW examined in univariate analyses (Supplementary Table S3), only monthly household income, the receipt of post-operative chemotherapy and radiotherapy, RTW self-efficacy, work conditions with long hours in sitting position, and QLQ-30 physical functioning retained significance in the final multiple linear regression model (Table 3). BCW with higher monthly household incomes of >US$3,850/month (<US$1,280: B = 68.82, 95%CI = 9.63–128.01, *p* = 0.022; US$1,280–3,850: B = 83.67, 95%CI = 38.57–128.77, *p* < 0.001), greater RTW self-efficacy (B = −53.77, 95%CI = −81.64 to −25.90, *p* < 0.001), more prolonged sitting at work (B = −47.52, 95%CI = −69.54 to −25.50, *p* < 0.001), and better physical functioning (B = −1.56, 95%CI = −2.67 to −0.46, *p* = 0.006) had taken shorter time to RTW. Delayed time to RTW was predicted by receipt of chemotherapy (B = 89.30, 95%CI = 46.30–132.30, *p* < 0.001) and/or radiotherapy (B = 61.72, 95%CI = 14.01–109.44, *p* = 0.011).

**Table 3:** Multiple forward linear regression on time to RTW (*n* = 371).

	***B***	**95%CI**	**β**	***t***	***p*-value**
**Monthly household income (US$)**					
US$ < 1,280	68.82	9.63–128.01	30.09	2.29	0.022^*^
US$ 1,280–3,850	83.67	38.57–128.77	22.92	3.65	< 0.001^**^
**US$** >**3,850**					
Chemotherapy	89.30	46.30–132.30	21.86	4.09	< 0.001^**^
Radiotherapy	61.72	14.01–109.44	24.25	2.55	0.011^*^
Return-to-work self-efficacy	−53.77	−81.64– −25.90	14.17	−3.80	< 0.001^**^
**Work condition**					
Frequent/long hours in sitting position	−47.52	−69.54– −25.50	11.19	−4.25	< 0.001^**^
QLQ-30 Physical functioning	−1.56	−2.67– −0.46	0.56	−2.78	0.006^*^
**Model statistics**					
*R* ^2^	0.28				
*P*-value	< 0.001^**^				

^*^p-value < 0.05; ^**^p-value < 0.001.

### 3.4 Work productivity loss at 12-month post-surgery

Patients successfully resuming work by 12 months following surgery (*n* = 161) reported an average 20.2% loss in work productivity. The final multiple linear regression model, which took into account significant covariates from univariate regression analyses (Supplementary Table S4), demonstrated that lower work satisfaction (B = −13.07, 95%CI = −18.79 to −7.35, *p* < 0.001), physically demanding work conditions (B = 11.48, 95%CI = 6.18–16.78, *p* < 0.001), higher QLQ-C30 role functioning (B = 0.52, 95%CI = 0.20–0.83, *p* = 0.002), and higher levels of pain (B = 0.75, 95%CI 0.43–1.08, *p* < 0.001) were all associated with greater work productivity loss at 12 months following surgery (Table 4).

**Table 4:** Multiple forward linear regression on work productivity loss at 12-month post-surgery (*n* = 161).

	**B**	**95%CI**	**β**	**t**	***p*-value**
Work satisfaction	−13.07	−18.79–−7.35	−0.55	−4.74	< 0.001^**^
**Work condition**					
Physically heavy work	11.48	6.18–16.78	0.47	4.50	< 0.001^**^
QLQ-C30 role functioning	0.52	0.20–0.83	0.55	0.32	0.002^*^
QLQ-C30 pain	0.75	0.43–1.08	0.74	4.79	< 0.001^**^
**Model statistics**					
R^2^	0.76				
*P*-value	< 0.001^**^				

^*^p-value < 0.05; ^**^p-value < 0.001.

### 3.5 Activity impairment at 12-month post-surgery

Patients reported an average 26.1% activity limitation at 12-month after surgery. The final multiple linear regression model of activity impairment, adjusted for significant covariates (Supplementary Table S5), showed that the receipt of hormonal therapy (B = 8.33, 95%CI 1.72–14.94, *p* = 0.014), poorer financial well-being (B = –0.47, 95%CI –0.78– –0.15, *p* = 0.004), a more B-IPQ negative cognitive representation of illness (B = 0.89, 95%CI = 0.46–1.32, *p* < 0.001), a higher level of QLQ-C30 insomnia (B = 0.10, 95%CI = 0.001–0.20, *p* = 0.048), and lower RTW self-efficacy (B = –6.09, 95%CI = –10.13– –2.06, *p* = 0.003) were all associated with greater activity impairment at 12 months after surgery (Table 5).

**Table 5:** Multiple forward linear regression on activity impairment at 12-month post-surgery (*n* = 227).

	***B***	**95%CI**	**β**	***t***	***p*-value**
Return-to-work self-efficacy	−6.09	−10.13–−2.06	−0.18	−2.97	0.003^*^
COST-financial well-being	−0.47	−0.78–−0.15	−0.19	−2.94	0.004^*^
Cognitive representations of illness	0.89	0.46–1.32	0.25	4.06	< 0.001^**^
Hormonal therapy	8.33	1.72–14.94	0.14	2.48	0.014^*^
QLQ-C30 insomnia	0.10	0.001–0.20	0.13	1.99	0.048^*^
**Model statistics**					
*R* ^2^	0.27				
*P*-value	< 0.001^**^				

^*^p-value < 0.05; ^**^p-value < 0.001.

## 4 Discussion

In this first longitudinally documented RTW study of Chinese BCW during the initial recovery stage following primary breast surgery, we observed upward trending RTW rates over time (12, 19) from 23.2% at 4-weeks to 68.2% at 12-month post-surgery. Approximately 2-in-3 BCW (68%) successfully RTW at the first year following surgery, comparable with the global mean RTW rates of 63.4% regardless of time since diagnosis from a systematic review of mixed cancer types (12), and within the 43%-93% BCW RTW range reported within 12 months of diagnosis elsewhere (18); both reviews being of predominantly western contexts. The observed RTW rate was relatively higher compared to other studies of Asian populations that reported rates of 21–59% (22, 24, 25). However, direct comparisons are challenging due to ambiguous or different RTW assessment timepoints used across these studies. Median time to RTW after BC in this study was ~183 days, comparable with BCW RTW studies in the early diagnostic and active treatment phases in developed countries such as France (155 days) (40) and the United Kingdom (210 days) (20), where statutory sick pay is available.

Adjuvant radiotherapy and chemotherapy, and physical impairment were associated with poor RTW outcomes, partially consistent with Mehnert's model (12). Radiotherapy can cause arm pain and limit movement. Chemotherapy-induced neuropathy or fatigue may impair to return to work (19, 21, 41). Poorer physical functioning is associated with RTW delay (17). Body image concerns, and societal expectations and stigmas surrounding BC can damage self-esteem and psychological well-being, resulting in the decision not to RTW (11, 18, 33).

In contrast, higher household income, white-collar work, favorable work conditions, and greater RTW self-efficacy promoted RTW outcomes. While higher household income can reflect employment (21, 26), it may also imply higher educational attainment, potentially higher job security, flexibility and less manual work, facilitating earlier resumption (7, 26). Similarly, favorable work conditions such as desk work, are less physically demanding, alleviating physical handicaps from BC treatments, smoothing return into the workforce (18, 21). Enhanced RTW self-efficacy also significantly contributed to early RTW, substantiating reports of lower self-efficacy among those not RTW in previous qualitative studies (27, 42). Poorer RTW self-efficacy reflects both perceived impaired working capability, and worries about potential workplace demands, impeding RTW (43). Job type influenced decisions on RTW but not duration of RTW. White-collar workers were more able to RTW after BC than were blue-collar workers (36). Blue-collar workers may face job loss and/or challenges in finding new employment due to both cancer and treatment impacts restricting more physically-skilled work (44, 45).

Work productivity loss in RTW BCW is common. Our sample reported a 20% reduction of their work productivity at 12-month post-surgery. Pain was weakly associated with work productivity loss, resonating with previous studies that unmanaged physical symptoms distress reduced working hours (46) and hindered work productivity (17, 47–49). Physically demanding work may exacerbate treatment-induced physical symptoms including pain, limiting work performance (12, 47, 50). Concern about physically demanding work causing upper-body lymphedema might further impair BCW productivity (49). Higher role functioning seemingly enhances work productivity loss, contradicting previous reports (51). Better role functioning may lead to taking on more responsibilities, generating more work-related stress and exhaustion (52), adversely affecting work performance (53). In contrast, job satisfaction was the sole protective predictor of work productivity among BCW. A supportive work environment and better interpersonal relationships at work facilitate work commitment and motivation, enhancing productivity (23, 54).

BCW commonly report cancer/treatment impacts on daily physical activities (55), affecting work-related outcomes (56). At 12-month post-surgery, BCW reporting reduced activity functioning had received hormonal therapy, experiencing severe sleep disturbance symptoms, lower RTW self-efficacy, poorer financial well-being, and negative cognitive representation of illness. Lymphedema is a common side effect of hormonal therapy (57) that cause swelling, pain, and limit range of motion in the affected arm or shoulder (58), impairing physical functioning and routine task performance (59). Sleep disturbance was inversely related to daytime alertness and quality of life (60), which might further worsen capacity to perform daily activities especially during daytime (61). Financial constraints restrict utilization of healthcare, social welfare and other resources (62). *Post-hoc* analysis suggested that patients experiencing greater financial difficulties tended not to have health insurance. Financial stress likely amplifies psychological distress in BCW, compounding cancer's impact on daily activities (62). Furthermore, negative illness perceptions were associated with greater activity impairment, perhaps attributable to lower perceived social support and well-being (63). Negative illness perception might also reflect residual symptoms which compound psychological distress (63), further impairing recovery.

### 4.1 Clinical implications

Supporting BCW in RTW and other work-related outcomes (5), particularly during early recovery stage after primary surgery is feasible and necessary. In Hong Kong, as elsewhere, few cancer rehabilitation resources exist or are outside routine clinical practice. A multifactorial RTW intervention would manage physical symptoms and side-effects but also address psychosocial (RTW self-efficacy), and work-environmental (promoting favorable work environments) issues at organizational or even policy levels, particularly for manual labor workers (5). RTW is context-specific, involving workplace policies, insurance protocol, culture, and resource availability—all of which could be regulated by legislation (5, 64). The Disability Discrimination Ordinance in Hong Kong guarantees people with chronic diseases including cancer, equal employment opportunities and reasonable workplace accommodation. However, challenges remain in effectively implementing and enforcing these regulations often due to employer intransigence (5, 64). In-house or external occupational health services, such as educational programs for employers might help. Occupational health professionals should also collaborate closely with occupational stakeholders to tailor organizational policy for creating supportive work environments and preventing work-related problems for BCW after RTW (64).

### 4.2 Study limitations

Study limitations include, first, BCW sampling was from government-funded public hospitals only. Being where most local patients received oncological care, the representativeness of our sample thus remains favorable. Second, an ideal control condition, such as RTW after acute coronary syndrome would clarify if the observed work-related outcomes were cancer-specific (65). Third, the COVID-19 pandemic may have influenced the RTW rate and duration (66). Furthermore, 31% of our sample had unspecified cancer staging (just 2% of participants with known disease stages had non-localized disease), but it is highly probable that almost all had early-stage cancer. Lastly, to minimize recall bias of self-reported RTW dates, averaged time to RTW was used in the analysis.

## 5 Conclusion

Early RTW after BC was relatively uncommon, with prolonged RTW delays, significant work productivity loss and activity impairment reported within the first year following surgery, highlighting the need for evidence-based rehabilitation interventions to support RTW and facilitate social reintegration (17). Work-related outcomes after BC evidenced multidimensional relationships with demographics, treatment-induced side effect and physical symptoms, financial well-being, work environment and satisfaction, RTW self-efficacy, and illness perception. These findings suggest that the development of a multifactorial, multidisciplinary RTW intervention might benefit Chinese BCW rehabilitation.

## Data availability statement

The raw data supporting the conclusions of this article will be made available by the authors, without undue reservation.

## Ethics statement

The studies involving humans were approved by HKU/HA HKW Institutional Review Board. The studies were conducted in accordance with the local legislation and institutional requirements. The participants provided their written informed consent to participate in this study.

## Author contributions

DN: Conceptualization, Data curation, Formal analysis, Investigation, Methodology, Project administration, Writing – original draft. SS: Data curation, Writing – review & editing. RF: Conceptualization, Supervision, Writing – review & editing. AM-T: Conceptualization, Validation, Writing – review & editing. AK: Resources, Writing – review & editing. DS: Resources, Writing – review & editing. LW: Resources, Writing – review & editing. SF: Resources, Writing – review & editing. OC: Resources, Writing – review & editing. DF: Writing – review & editing. SC: Resources, Writing – review & editing. AM: Writing – review & editing. WS: Writing – review & editing. WL: Conceptualization, Funding acquisition, Methodology, Supervision, Writing – review & editing.
