# Supplementary material for: Return to work, work productivity loss and activity impairment in Chinese breast cancer survivors 12-month post-surgery: a longitudinal study
Source: Front Public Health. 2024 Feb 23;12:1340920. doi: 10.3389/fpubh.2024.1340920 (PMC10920332; doi:10.3389/fpubh.2024.1340920)
Supplement: Supplementary file 1 [file Table_1.DOCX]

Supplementary Material

Return to work, work productivity loss and activity impairment in Chinese breast cancer survivors 12-month post-surgery: a longitudinal study

**Danielle Wing Lam Ng^1,2^, Serana Chun Yee So^1,2^, Richard Fielding^1,2^, Anja Mehnert-Theuerkauf^3^, Ava Kwong^4^, Dacita Suen^4^, Ling Wong^5^, Sara Wai Wun Fung^6^, Oi Kwan Chun^6^, Daniel Fong^7^, Sharon Chan^8^, Alex Molasiotis^9,10^, Winnie So^11^, Wendy Wing Tak Lam^1,2*^**

*** Correspondence:** Wendy Wing Tak Lam: [wwtlam@hku.hk](mailto:wwtlam@hku.hk)

# Supplementary Figures and Tables

## Supplementary Tables

Table S1. Summary of demographic and clinical characteristics among participants being included in and being excluded from analysis

|  | Participants included in analysis (%) | Participants excluded from analysis (%) | t/X^2^ | p-value |  |  |
| --- | --- | --- | --- | --- | --- | --- |
|  | n = 371 | n = 7 |  |  |  |  |
| Demographic characteristics |  |  |  |  |  |  |
| Age at diagnosis (year) ± standard deviation (SD) | 52.62±8.22 | 49.14±9.70 | 0.52 | 0.60 |  |  |
| Time since cancer diagnosis (months) ± standard deviation (SD) | 7.18±11.04 | 5.00±5.97 | 1.11 | 0.38 |  |  |
| Marital status |  |  | 0.026 | 0.87 |  |  |
| Married/ cohabited | 222 (60.2) | 4 (57.1) |  |  |  |  |
| Single/ divorced/ separated/ widowed | 147 (39.8) | 3 (42.9) |  |  |  |  |
| Educational level |  |  | 1.17 | 0.28 |  |  |
| No formal/ primary education | 52 (14.1) | 2 (38.6) |  |  |  |  |
| Secondary/ tertiary | 317 (85.9) | 5 (71.4) |  |  |  |  |
| Job title |  |  | 1.63 | 0.44 |  |  |
| White collar | 140 (37.7) | 1 (14.3) |  |  |  |  |
| Blue collar | 159 (42.9) | 51 (57.1) |  |  |  |  |
| Professional, manager and self-employed | 72 (19.4) | 2 (28.6) |  |  |  |  |
| Monthly household income (US$) |  |  | 0.76 | 0.68 |  |  |
| US$ <1,280 | 62 (16.9) | 2 (28.6) |  |  |  |  |
| US$ 1,280 – 3,850 | 150 (41.8) | 3 (42.9) |  |  |  |  |
| US$ >3,850 | 147 (40.9) | 2 (28.6) |  |  |  |  |
| Clinical characteristics |  |  |  |  |  |  |
| Stage |  |  | 270.10 | <0.001** |  |  |
| Stage 0 | 31 (8.4) | 0 (0%) |  |  |  |  |
| Stage I | 126 (34.0) | 0 (0%) |  |  |  |  |
| Stage II | 66 (17.8) | 0 (0%) |  |  |  |  |
| Stage III | 32 (8.6) | 1 (14.3) |  |  |  |  |
| Stage IV | 0 (0%) | 5 (71.4) |  |  |  |  |
| Unknown | 116 (31.1) | 1 (14.3) |  |  |  |  |
| Surgery type |  |  | 0.85 | 0.36 |  |  |
| Breast conserving surgery and others | 165 (46.1) | 2 (28.6) |  |  |  |  |
| Mastectomy or plus reconstruction | 193 (53.9) | 5 (71.4) |  |  |  |  |
| Post-operative treatment |  |  |  |  |  |  |
| Chemotherapy | 141 (38.0) | 4 (57.1) | 1.06 | 0.30 |  |  |
| Radiotherapy | 98 (26.4) | 2 (28.6) | 0.016 | 0.90 |  |  |
| Target therapy | 71 (19.9) | 3 (42.9) | 2.22 | 0.14 |  |  |
| Hormonal therapy | 242 (65.2) | 5 (71.4) | 0.12 | 0.73 |  |  |
| No Post-operative treatment | 72 (19.4) | 1 (14.3) | 0.12 | 0.73 |  |  |
| Baseline predictors |  |  |  |  |  |  |
| Return-to-work self-efficacy (mean ± SD) | 4.03±0.75 | 3.01±0.37 | 0.42 | 0.66 |  |  |
| COST-Financial well-being (mean ± SD) | 22.75±10.56 | 18.43±9.54 | 1.07 | 0.28 |  |  |
| IPQ-Illness perception (mean ± SD) |  |  |  |  |  |  |
| Cognitive representations of illness | 19.17±7.12 | 22.14±3.85 | -1.10 | 0.27 |  |  |
| Emotional representations of illness | 10.69±4.94 | 13.71±5.35 | -1.60 | 0.11 |  |  |
| Work satisfaction (mean ± SD) | 4.97±1.08 | 5.15±1.16 | -0.43 | 0.67 |  |  |
| Job strain (mean ± SD) | 2.41±0.84 | 1.83±0.86 | 1.83 | 0.069 |  |  |
| Work condition (mean ± SD) |  |  |  |  |  |  |
| Physically heavy work | 2.76±0.94 | 2.57±0.79 | 0.54 | 0.59 |  |  |
| Incorrect one-sided posture | 2.71±0.88 | 2.29±0.95 | 1.27 | 0.21 |  |  |
| Frequent/long hours in sitting position | 2.83±0.96 | 2.57±1.13 | 0.71 | 0.48 |  |  |
| Wetness, coldness, and draft | 2.31±0.80 | 2.57±0.54 | -0.87 | 0.38 |  |  |
| Rationalization and restructuring | 2.12±0.75 | 2.00±0.82 | 0.43 | 0.67 |  |  |
| Implementation of new technologies | 2.25±0.83 | 1.71±0.49 | 1.70 | 0.09 |  |  |
| Excessive demands | 2.66±0.79 | 2.43±0.79 | 0.78 | 0.44 |  |  |
| Satisfying work nature | 3.20±0.64 | 2.86±0.69 | 1.41 | 0.16 |  |  |
| QLQ-C30 Global health status (mean ± SD) | 61.10±17.92 | 64.29±14.20 | -0.47 | 0.64 |  |  |
| QLQ-C30 Functional scales (mean ± SD) |  |  |  |  |  |  |
| Physical functioning | 74.00±19.49 | 79.05±19.79 | -0.68 | 0.50 |  |  |
| Role functioning | 67.34±27.08 | 69.95±26.23 | -0.17 | 0.87 |  |  |
| Emotional functioning | 72.06±22.64 | 69.05±29.15 | 0.35 | 0.72 |  |  |
| Cognitive functioning | 76.01±22.18 | 71.43±29.99 | 0.54 | 0.59 |  |  |
| Social functioning | 75.11±25.57 | 80.95±31.07 | -0.60 | 0.55 |  |  |
| QLQ-C30 Symptoms scales (mean ± SD) |  |  |  |  |  |  |
| Fatigue | 35.34±24.16 | 33.33±28.69 | 0.22 | 0.83 |  |  |
| Nausea | 5.39±12.87 | 0±0 | 8.06 | <0.001** |  |  |
| Pain | 32.35±24.70 | 30.95±17.82 | 0.15 | 0.88 |  |  |
| Dyspnea | 13.75±23.37 | 9.52±16.27 | 0.48 | 0.64 |  |  |
| Insomnia | 32.35±32.45 | 14.29±17.82 | 1.47 | 0.14 |  |  |
| Appetite loss | 13.84±22.47 | 5.76±12.60 | 1.85 | 0.11 |  |  |
| Constipation | 6.83±14.95 | 0±0 | 8.80 | <0.001** |  |  |
| Diarrhea | 6.68±17.99 | 9.52±25.20 | -0.41 | 0.68 |  |  |
| Financial difficulties | 31.08±32.52 | 33.33±38.49 | -0.18 | 0.86 |  |  |
| QLQ-BC23 Functional scales (mean ± SD) |  |  |  |  |  |  |
| Body image | 78.46±25.62 | 77.38±27.09 | 0.11 | 0.91 |  |  |
| Sexual functioning | 92.73±14.43 | 90.48±16.27 | 0.41 | 0.68 |  |  |
| Sexual enjoyment | 61.11±23.12 | 66.67±0 | -0.34 | 0.74 |  |  |
| Future perspective | 47.57±30.08 | 38.10±29.99 | 0.83 | 0.41 |  |  |
| QLQ-BC23 Symptoms scales (mean ± SD) |  |  |  |  |  |  |
| Systematic therapy side effect | 15.94±15.13 | 8.84±8.44 | 1.23 | 0.22 |  |  |
| Breast symptoms | 20.97±20.30 | 14.29±10.45 | 0.87 | 0.39 |  |  |
| Arm symptoms | 26.68±22.17 | 25.40±15.33 | 0.15 | 0.88 |  |  |
| Upset by hair loss | 38.61±32.62 | - | NA | NA |  |  |
| HADS Anxiety (mean ± SD) | 5.40±3.91 | 6.57±6.83 | -0.45 | 0.67 |  |  |
| HADS Depression (mean ± SD) | 4.71±3.80 | 6.00±5.45 | -0.62 | 0.56 |  |  |
| Note: FU1= Follow-up 1 4-month post-baseline; FU2= Follow-up 2 6-month post-baseline; FU3= Follow-up 3 12-month post-baseline; N/A= not applicable; *p-value<0.05; **p-value<0.001. | | | | |  |  |

Table S2. Univariate Cox regression on RTW status (n=371)

|  | β | SE | HR | 95%CI | p-value |  |
| --- | --- | --- | --- | --- | --- | --- |
| Demographic characteristics |  |  |  |  |  |  |
| Age at diagnosis (year) | -0.005 | 0.008 | 1.00 | 0.98-1.01 | 0.50 |  |
| Time since cancer diagnosis (months) | 0.002 | 0.006 | 1.00 | 0.99-1.01 | 0.78 |  |
| Marital status |  |  |  |  |  |  |
| Married/ cohabited | -0.16 | 0.13 | 0.85 | 0.66-1.10 | 0.22 |  |
| Single/ divorced/ separated/ widowed (ref) |  |  |  |  |  |  |
| Educational level |  |  |  |  |  |  |
| No formal/ primary education (ref) |  |  |  |  |  |  |
| Secondary/ tertiary | 0.27 | 0.20 | 1.31 | 0.89-1.91 | 0.17 |  |
| Job title |  |  |  |  | <0.001** |  |
| Blue collar (ref) |  |  |  |  |  |  |
| White collar | 0.85 | 0.15 | 2.37 | 1.77-3.18 | <0.001** |  |
| Professional, manager and self-employed | 0.89 | 0.18 | 2.44 | 1.73-3.44 | <0.001** |  |
| Monthly household income (US$) |  |  |  |  | <0.001** |  |
| US$ <1,280 |  |  |  |  |  |  |
| US$ 1,280 – 3,850 | 0.16 | 0.21 | 1.17 | 0.78-1.76 | 0.45 |  |
| US$ >3,850 | 0.76 | 0.20 | 2.14 | 1.44-3.19 | <0.001** |  |
| Clinical characteristics |  |  |  |  |  |  |
| Stage |  |  |  |  | 0.011* |  |
| Stage 0 (ref) |  |  |  |  |  |  |
| Stage I | -0.45 | 0.23 | 0.64 | 0.41-1.00 | 0.049* |  |
| Stage II | -0.77 | 0.26 | 0.46 | 0.28-0.78 | 0.004* |  |
| Stage III | -0.45 | 0.29 | 0.64 | 0.36-1.13 | 0.12 |  |
| Unknown | -0.17 | 0.23 | 0.85 | 0.54-1.32 | 0.47 |  |
| Surgery type |  |  |  |  | 0.013* |  |
| Breast conserving surgery (ref) |  |  |  |  |  |  |
| Mastectomy or plus reconstruction | -0.33 | 0.13 | 0.72 | 0.56-0.93 | 0.013* |  |
| Post-operative treatment |  |  |  |  |  |  |
| Chemotherapy | -0.70 | 0.14 | 0.50 | 0.38-0,65 | <0.001** |  |
| Radiotherapy | -0.60 | 0.16 | 0.55 | 0.40-0.75 | <0.001** |  |
| Target therapy | -0.65 | 0.18 | 0.52 | 0.37-0.75 | <0.001** |  |
| Hormonal therapy | -0.012 | 0.14 | 0.99 | 0.76-1.29 | 0.93 |  |
| No Post-operative treatment | 0.49 | 0.16 | 1.63 | 1.20-2.21 | 0.002* |  |
| Baseline predictors |  |  |  |  |  |  |
| Return-to-work self-efficacy | 0.39 | 0.071 | 1.48 | 1.29-1.71 | <0.001** |  |
| COST-Financial well-being | 0.34 | 0.006 | 1.04 | 1.02-1.05 | <0.001** |  |
| IPQ-Illness perception |  |  |  |  |  |  |
| Cognitive representations of illness | -0.027 | 0.009 | 0.97 | 0.96-0.99 | 0.002* |  |
| Emotional representations of illness | -0.032 | 0.013 | 0.97 | 0.94-0.99 | 0.012* |  |
| Work satisfaction | 0.25 | 0.061 | 1.28 | 1.14-1.44 | <0.001* |  |
| Job strain | -0.007 | 0.077 | 0.99 | 0.85-1.16 | 0.92 |  |
| Work condition |  |  |  |  |  |  |
| Physically heavy work | -0.31 | 0.067 | 0.73 | 0.65-0.84 | <0.001** |  |
| Incorrect one-sided posture | -0.18 | 0.072 | 0.84 | 0.72-0.96 | 0.012* |  |
| Frequent/long hours in sitting position | 0.38 | 0.069 | 1.46 | 1.27-1.67 | <0.001** |  |
| Wetness, coldness, and draft | -0.072 | 0.079 | 0.93 | 0.80-1.09 | 0.36 |  |
| Rationalization and restructuring | 0.093 | 0.087 | 1.10 | 0.93-1.30 | 0.29 |  |
| Implementation of new technologies | 0.12 | 0.079 | 1.14 | 0.98-1.33 | 0.094 |  |
| Excessive demands | -0.078 | 0.082 | 0.93 | 0.79-1.09 | 0.34 |  |
| Satisfying work nature | 0.22 | 0.10 | 1.24 | 1.01-1.52 | 0.038* |  |
| QLQ-C30 Global health status | 0.010 | 0.004 | 1.01 | 1.00-1.02 | 0.005* |  |
| QLQ-C30 Functional scales |  |  |  |  |  |  |
| Physical functioning | 0.019 | 0.004 | 1.02 | 1.01-1.03 | <0.001** |  |
| Role functioning | 0.010 | 0.003 | 1.01 | 1.01-1.02 | <0.001** |  |
| Emotional functioning | 0.009 | 0.003 | 1.01 | 1,00-1.02 | 0.002* |  |
| Cognitive functioning | 0.004 | 0.003 | 1.00 | 0.99-1.01 | 0.14 |  |
| Social functioning | 0.002 | 0.003 | 1.00 | 0.99-1.01 | 0.52 |  |
| QLQ-C30 Symptoms scales |  |  |  |  |  |  |
| Fatigue | -0.010 | 0.003 | 0.99 | 0.98-0.99 | <0.001** |  |
| Nausea | -0.008 | 0.005 | 0.99 | 0.98-1.00 | 0.15 |  |
| Pain | -0.012 | 0.003 | 0.99 | 0.98-0.99 | <0.001** |  |
| Dyspnea | -0.004 | 0.003 | 1.00 | 0.99-1.00 | 0.14 |  |
| Insomnia | -0.005 | 0.002 | 0.99 | 0.99-0.99 | 0.010* |  |
| Appetite loss | -0.003 | 0.003 | 1.00 | 0.99-1.00 | 0.31 |  |
| Constipation | 0.010 | 0.004 | 1.01 | 1.00-1.02 | 0.018* |  |
| Diarrhea | 0.00 | 0.004 | 1.00 | 0.99-1.01 | 0.99 |  |
| Financial difficulties | -0.010 | 0.002 | 0.99 | 0.98-0.99 | <0.001** |  |
| QLQ-BC23 Functional scales |  |  |  |  |  |  |
| Body image | 0.005 | 0.003 | 1.01 | 1.00-1.01 | 0.057 |  |
| Sexual functioning | -0.012 | 0.004 | 0.99 | 0.98-0.99 | 0.005* |  |
| Sexual enjoyment | -0.006 | 0.007 | 0.99 | 0.98-1.01 | 0.36 |  |
| Future perspective | 0.004 | 0.002 | 1.00 | 0.99-1.01 | 0.091 |  |
| QLQ-BC23 Symptoms scales |  |  |  |  |  |  |
| Systematic therapy side effect | -0.004 | 0.004 | 1.00 | 0.99-1.01 | 0.40 |  |
| Breast symptoms | -0.005 | 0.003 | 1.00 | 0.99-1.00 | 0.16 |  |
| Arm symptoms | -0.012 | 0.003 | 0.99 | 0.98-0.99 | <0.001** |  |
| Upset by hair loss | -0.001 | 0.004 | 1.00 | 0.99-1.01 | 0.84 |  |
| HADS Anxiety | -0.046 | 0.017 | 0.96 | 0.92-0.99 | 0.007* |  |
| HADS Depression | -0.059 | 0.018 | 0.94 | 0.91-0.98 | 0.001* |  |

Note: *p-value<0.05; **p-value<0.001.

Table S3. Univariate analysis on time to RTW (n=371)

|  | F/r | p-value |  |
| --- | --- | --- | --- |
| Demographic characteristics |  |  |  |
| Age at diagnosis (year) | - | NS |  |
| Time since cancer diagnosis (months) | - | NS |  |
| Marital status | - | NS |  |
| Married/ cohabited |  |  |  |
| Single/ divorced/ separated/ widowed (ref) |  |  |  |
| Educational level | - | NS |  |
| No formal/ primary education (ref) |  |  |  |
| Secondary/ tertiary |  |  |  |
| Job title | 24.02 | <0.001** |  |
| Blue collar (ref) |  |  |  |
| White collar |  |  |  |
| Professional, manager and self-employed |  |  |  |
| Monthly household income (US$) | 11.63 | <0.001** |  |
| US$ <1,280 |  |  |  |
| US$ 1,280 – 3,850 |  |  |  |
| US$ >3,850 |  |  |  |
| Clinical characteristics |  |  |  |
| Stage | 3.67 | 0.006* |  |
| Stage 0 (ref) |  |  |  |
| Stage I |  |  |  |
| Stage II |  |  |  |
| Stage III |  |  |  |
| Unknown |  |  |  |
| Surgery type | 6.68 | 0.010* |  |
| Breast conserving surgery (ref) |  |  |  |
| Mastectomy or plus reconstruction |  |  |  |
| Post-operative treatment |  |  |  |
| Chemotherapy | 37.18 | <0.001** |  |
| Radiotherapy | 19.56 | <0.001** |  |
| Target therapy | 17.85 | <0.001** |  |
| Hormonal therapy | - | NS |  |
| No Post-operative treatment | 10.13 | 0.002* |  |
| Baseline predictors |  |  |  |
| Return-to-work self-efficacy | -0.30 | <0.001** |  |
| COST-Financial well-being | -0.30 | <0.001** |  |
| IPQ-Illness perception |  |  |  |
| Cognitive representations of illness | 0.16 | 0.003* |  |
| Emotional representations of illness | 0.13 | 0.016* |  |
| Work satisfaction | -0.22 | <.001** |  |
| Job strain | - | NS |  |
| Work condition |  |  |  |
| Physically heavy work | 0.24 | <0.001** |  |
| Incorrect one-sided posture | 0.13 | 0.015* |  |
| Frequent/long hours in sitting position | -0.29 | <0.001** |  |
| Wetness, coldness, and draft | - | NS |  |
| Rationalization and restructuring | - | NS |  |
| Implementation of new technologies | - | NS |  |
| Excessive demands | - | NS |  |
| Satisfying work nature | -0.10 | 0.040* |  |
| QLQ-C30 Global health status | -0.14 | 0.006* |  |
| QLQ-C30 Functional scales |  |  |  |
| Physical functioning | -0.28 | <0.001** |  |
| Role functioning | -0.21 | <0.001** |  |
| Emotional functioning | -0.16 | 0.002* |  |
| Cognitive functioning | - | NS |  |
| Social functioning | - | NS |  |
| QLQ-C30 Symptoms scales |  |  |  |
| Fatigue | 0.20 | <0.001 |  |
| Nausea | - | NS |  |
| Pain | 0.22 | <0.001 |  |
| Dyspnea | - | NS |  |
| Insomnia | 0.13 | 0.012* |  |
| Appetite loss | - | NS |  |
| Constipation | -0.11 | 0.044* |  |
| Diarrhea | - | NS |  |
| Financial difficulties | 0.25 | <0.001** |  |
| QLQ-BC23 Functional scales |  |  |  |
| Body image | -0.104 | 0.048* |  |
| Sexual functioning | 0.13 | 0.011* |  |
| Sexual enjoyment | - | NS |  |
| Future perspective | - | NS |  |
| QLQ-BC23 Symptoms scales |  |  |  |
| Systematic therapy side effect | - | NS |  |
| Breast symptoms | - | NS |  |
| Arm symptoms | 0.21 | <0.001** |  |
| Upset by hair loss | - | NS |  |
| HADS Anxiety | 0.13 | 0.013* |  |
| HADS Depression | 0.17 | 0.002* |  |

Note: *p-value<0.05; **p-value<0.001.

Table S4. Univariate analysis on work productivity loss at 12-month post-surgery (n=161)

|  | F/r | p-value |  |
| --- | --- | --- | --- |
| Demographic characteristics |  |  |  |
| Age at diagnosis (year) | - | NS |  |
| Time since cancer diagnosis (months) | - | NS |  |
| Marital status | 4.46 | 0.036 |  |
| Married/ cohabited |  |  |  |
| Single/ divorced/ separated/ widowed (ref) |  |  |  |
| Educational level | - | NS |  |
| No formal/ primary education (ref) |  |  |  |
| Secondary/ tertiary |  |  |  |
| Job title | - | NS |  |
| Blue collar (ref) |  |  |  |
| White collar |  |  |  |
| Professional, manager and self-employed |  |  |  |
| Monthly household income (US$) |  |  |  |
| US$ <1,280 (ref) | - | NS |  |
| US$ 1,280 – 3,850 |  |  |  |
| US$ >3,850 |  |  |  |
| Clinical characteristics |  |  |  |
| Stage | 3.20 | 0.015* |  |
| Stage 0 (ref) |  |  |  |
| Stage I |  |  |  |
| Stage II |  |  |  |
| Stage III |  |  |  |
| Surgery type | - | NS |  |
| Breast conserving surgery (ref) |  |  |  |
| Mastectomy or plus reconstruction |  |  |  |
| Post-operative treatment |  |  |  |
| Chemotherapy | 5.05 | 0.026* |  |
| Radiotherapy | - | NS |  |
| Target therapy | 6.36 | 0.013* |  |
| Hormonal therapy | 5.74 | 0.018* |  |
| No Post-operative treatment | 11.70 | <0.001** |  |
| Baseline predictors |  |  |  |
| Return-to-work self-efficacy | -0.17 | 0.030* |  |
| COST-Financial well-being | -0.31 | <0.001* |  |
| IPQ-Illness perception |  |  |  |
| Cognitive representations of illness | 0.32 | <0.001** |  |
| Emotional representations of illness | 0.27 | <0.001** |  |
| Work satisfaction | -0.20 | 0.010* |  |
| Job strain | 0.17 | 0.034* |  |
| Work condition |  |  |  |
| Physically heavy work | 0.17 | 0.030* |  |
| Incorrect one-sided posture | - | NS |  |
| Frequent/long hours in sitting position | - | NS |  |
| Wetness, coldness, and draft | - | NS |  |
| Rationalization and restructuring | - | NS |  |
| Implementation of new technologies | - | NS |  |
| Excessive demands | - | NS |  |
| Satisfying work nature | - | NS |  |
| QLQ-C30 Global health status | -0.29 | <0.001** |  |
| QLQ-C30 Functional scales |  |  |  |
| Physical functioning | -0.30 | <0.001** |  |
| Role functioning | -0.31 | <0.001** |  |
| Emotional functioning | -0.21 | <0.001** |  |
| Cognitive functioning | - | NS |  |
| Social functioning | - | NS |  |
| QLQ-C30 Symptoms scales |  |  |  |
| Fatigue | 0.32 | <0.001** |  |
| Nausea | - | NS |  |
| Pain | 0.30 | <0.001** |  |
| Dyspnea | 0.24 | 0.003* |  |
| Insomnia | 0.19 | 0.017* |  |
| Appetite loss | 0.16 | 0.038 |  |
| Constipation | 0.20 | 0.012* |  |
| Diarrhea | - | NS |  |
| Financial difficulties | 0.22 | 0.004* |  |
| QLQ-BC23 Functional scales |  |  |  |
| Body image | -0.18 | 0.020* |  |
| Sexual functioning | - | NS |  |
| Sexual enjoyment | -0.44 | 0.020* |  |
| Future perspective | -0.25 | 0.001* |  |
| QLQ-BC23 Symptoms scales |  |  |  |
| Systematic therapy side effect | 0.28 | <0.001** |  |
| Breast symptoms | 0.17 | 0.036* |  |
| Arm symptoms | 0.28 | <0.001** |  |
| Upset by hair loss | - | NS |  |
| HADS Anxiety | 0.25 | 0.001* |  |
| HADS Depression | 0.33 | <0.001** |  |

Note: *p-value<0.05; **p-value<0.001.

Table S5. Univariate analysis on activity impairment at 12-month post-surgery (n=242)

|  | F/r | p-value |  |
| --- | --- | --- | --- |
| Demographic characteristics |  |  |  |
| Age at diagnosis (year) | - | NS |  |
| Time since cancer diagnosis (month) | - | NS |  |
| Marital status | - | NS |  |
| Married/ cohabited |  |  |  |
| Single/ divorced/ separated/ widowed (ref) |  |  |  |
| Educational level | - | NS |  |
| No formal/ primary education (ref) |  |  |  |
| Secondary/ tertiary |  |  |  |
| Job title | - | NS |  |
| Blue collar (ref) |  |  |  |
| White collar |  |  |  |
| Professional, manager and self-employed |  |  |  |
| Monthly household income (US$) |  |  |  |
| US$ <1,280 (ref) | - | NS |  |
| US$ 1,280 – 3,850 |  |  |  |
| US$ >3,850 |  |  |  |
| Clinical characteristics |  |  |  |
| Stage | 2.96 | 0.021* |  |
| Stage 0 (ref) |  |  |  |
| Stage I |  |  |  |
| Stage II |  |  |  |
| Stage III |  |  |  |
| Surgery type | 5.15 | 0.024* |  |
| Breast conserving surgery (ref) |  |  |  |
| Mastectomy or plus reconstruction |  |  |  |
| Post-operative treatment |  |  |  |
| Chemotherapy | 7.84 | 0.006* |  |
| Radiotherapy | 4.58 | 0.033* |  |
| Target therapy | - | NS |  |
| Hormonal therapy | 7.80 | 0.006* |  |
| No Post-operative treatment | 9.29 | 0.003* |  |
| Baseline predictors |  |  |  |
| Return-to-work self-efficacy | -0.28 | <0.001** |  |
| COST-Financial well-being | -0.37 | <0.001* |  |
| IPQ-Illness perception |  |  |  |
| Cognitive representations of illness | 0.35 | <0.001** |  |
| Emotional representations of illness | 0.29 | <0.001** |  |
| Work satisfaction | -0.27 | <0.001** |  |
| Job strain | 0.15 | 0.023* |  |
| Work condition |  |  |  |
| Physically heavy work | 0.14 | 0.032* |  |
| Incorrect one-sided posture | - | NS |  |
| Frequent/long hours in sitting position | - | NS |  |
| Wetness, coldness, and draft | - | NS |  |
| Rationalization and restructuring | - | NS |  |
| Implementation of new technologies | - | NS |  |
| Excessive demands | - | NS |  |
| Satisfying work nature | - | NS |  |
| QLQ-C30 Global health status | -0.31 | <0.001** |  |
| QLQ-C30 Functional scales |  |  |  |
| Physical functioning | -0.32 | <0.001** |  |
| Role functioning | -0.28 | <0.001** |  |
| Emotional functioning | -0.29 | <0.001** |  |
| Cognitive functioning | -0.22 | <0.001** |  |
| Social functioning | -0.16 | 0.013* |  |
| QLQ-C30 Symptoms scales |  |  |  |
| Fatigue | 0.36 | <0.001** |  |
| Nausea | 0.15 | 0.017* |  |
| Pain | 0.32 | <0.001** |  |
| Dyspnea | 0.21 | <0.001** |  |
| Insomnia | 0.31 | <0.001** |  |
| Appetite loss | 0.13 | 0.041* |  |
| Constipation | - | NS |  |
| Diarrhea | - | NS |  |
| Financial difficulties | 0.25 | <0.001** |  |
| QLQ-BC23 Functional scales |  |  |  |
| Body image | -0.15 | 0.024* |  |
| Sexual functioning | - | NS |  |
| Sexual enjoyment | - | NS |  |
| Future perspective | -0.28 | <0.001** |  |
| QLQ-BC23 Symptoms scales |  |  |  |
| Systematic therapy side effect | 0.20 | 0.002* |  |
| Breast symptoms | 0.13 | 0.048* |  |
| Arm symptoms | 0.30 | <0.001** |  |
| Upset by hair loss | - | NS |  |
| HADS Anxiety | 0.31 | <0.001** |  |
| HADS Depression | 0.36 | <0.001** |  |

Note: *p-value<0.05; **p-value<0.001.

## Supplementary Figures


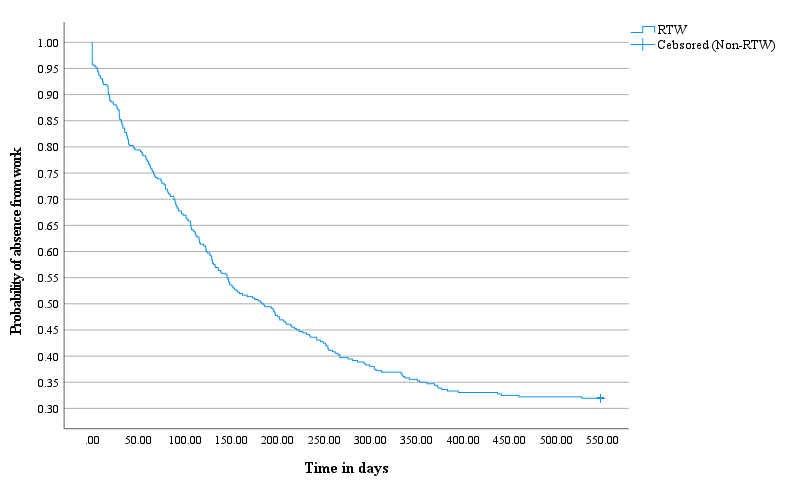


Mean time to RTW= 261 days

Median time to RTW= 183 days

66 days

183 days

Figure S1. The probability of absence from work over time.
